# Meta-Analysis of Environmental Impacts on Nitrous Oxide Release in Response to N Amendment

**DOI:** 10.3389/fmicb.2012.00272

**Published:** 2012-07-31

**Authors:** Emma L. Aronson, Steven D. Allison

**Affiliations:** ^1^Department of Ecology and Evolutionary Biology, University of California IrvineIrvine, CA, USA; ^2^Department of Earth System Science, University of California IrvineIrvine, CA, USA

**Keywords:** denitrification, meta-analysis, N amendment, N deposition, N_2_O, nitrification, nitrous oxide

## Abstract

Atmospheric nitrous oxide (N_2_O) accounts for approximately 5% of the global greenhouse effect and destroys stratospheric ozone. Soils are the most important source of N_2_O, which is produced during nitrification and denitrification. To assess the impact of environmental variables and ecosystems on N_2_O flux, we performed a meta-analysis comparing N_2_O flux in N amended and matched control plots in non-agricultural soils. We found that N_2_O release increased with N amendment in the short term. Although there were few studies in shrubland, this ecosystem showed the greatest response. The N_2_O response to N amendment was greater in year-round studies and in studies with more measurements, but lower in longer studies. The N_2_O response was greater at higher latitudes and precipitation rates. We also observed an unexpected 55% decline in the N_2_O response to N amendment over the 23 years covered by the studies. This pattern may reflect a suppression of the N_2_O response from long-term N deposition accumulation, particularly in temperate regions. Although short term increases in reactive N entering natural systems may cause positive feedbacks to the release of N_2_O, this effect may diminish over time in locations with high rates of N deposition.

## Introduction

Human activities have doubled the amount of nitrogen (N) entering soils, primarily through fossil fuel combustion and the application of N fertilizer to agricultural land (Schlesinger, 2009). Increased N inputs and cycling have led to increased N deposition on natural ecosystems (Galloway et al., 2008), stimulating plant growth, and altering soil microbial responses (Lu et al., 2011). Increased soil N availability can also stimulate losses of trace gases, such as nitrous oxide (N_2_O), which accounts for approximately 5% of global greenhouse gas forcing. Oxides of N derived from N_2_O can also react with the Earth’s protective stratospheric ozone layer and expose the surface to harmful UV rays from the sun (Intergovernmental Panel on Climate Change, 2007). Therefore, predictions of climate feedbacks involving N deposition must account for N_2_O release (Zaehle et al., 2011).

The main sources of atmospheric N_2_O are the reduction of nitrate through denitrification, and the oxidation of ammonia to nitrite, and further to nitrate during nitrification (Pathak, 1999). Soil type, oxygen status, moisture, temperature, carbon (C), and N status, as well as N amendment to the soil, can influence both of these processes and subsequent N_2_O release (Pathak, 1999; Burgin and Groffman, 2012). In agricultural systems, fertilizer type, fertilizer amount, and crop type are important factors determining the rate of N_2_O release (Bouwman et al., 2002). In non-agricultural systems, most reactive N enters the system through atmospheric deposition. For example, elevated N deposition can increase N_2_O release by up to fivefold in forest soils (Butterbach-Bahl et al., 1998).

Most N_2_O research has focused on agricultural soils, which generally release more N_2_O than non-agricultural soils (Reay et al., 2012). However, non-agricultural soil sources are increasing in importance as perturbation of the N cycle continues (Gruber and Galloway, 2008). In order to address the response of non-agricultural systems to greater N loads, we collected published comparisons of soil N_2_O release in fertilized and unfertilized soils in upland ecosystems. This analysis contrasts with previous meta-analyses that have focused on the fertilization response of plant N (Lu et al., 2011), and C pools and fluxes (Treseder, 2008; Liu and Greaver, 2009; Aronson and Helliker, 2010; Janssens et al., 2010). We focus on non-wetland and non-agricultural soils, since far less is known about the response of these soils to N amendments.

We hypothesized that reactive N amendment would significantly increase N_2_O release across studies (e.g., Barnard et al., 2005). In addition, we hypothesized that the response of N_2_O release to N amendment would increase with level of N amendment and daily precipitation. The precipitation hypothesis was based on data showing increased N_2_O release from denitrification following precipitation and irrigation (Freney et al., 1978; Duxbury et al., 1982; Pathak, 1999). We therefore hypothesized that more arid systems, such as deserts, savannas, grasslands, and shrublands would show the lowest N_2_O response to N amendment, whereas forests, particularly in the humid tropics, would show the greatest response.

## Materials and Methods

### Data sources

Nitrous oxide production data were extracted from published studies containing matched N amendment and control treatments (Appendix). The studies included in this meta-analysis were performed in non-agricultural, non-wetland ecosystems. All studies performed field flux measurements using chambers, although the chamber type and N_2_O quantification methods varied. All the original data are extracted from text, figures, and tables in the published papers, as in Aronson and Helliker (2010). The studies were located using ISI Web of Knowledge with the search terms: “nitrous oxide” and “release” or “flux” and “fertilization” or “nitrogen” and “amendment,” “addition” or “deposition.” The resulting database consisted of 99 entries from 33 papers, each containing a single added N versus control comparison (Supplementary Material).

There were multiple comparisons from many studies due to different levels of N treatment or multi-factorial designs. When multiple N amendment levels were used, the average flux from un-amended plots was used for the control in all comparisons. In multi-factorial experiments, comparisons were made between N treatment and N control plots that had received the same set of crossed treatments.

Ancillary information from each data source included: latitude, average annual temperature, average daily precipitation, start year, study duration, number of measurements, ecosystem type, season(s) studied, form(s) and amount of N used, and gas flux data collection method. We also recorded whether the plots had been fertilized previously or only for the duration of the gas flux study. The forms of fertilizer used were ammonium, nitrate, urea, and unknown (when the type of N was not given). If the temperature and precipitation during the study were not given in the publication, we used climate data from the NOAA Climate Data Online system from 1 day before until the end of the study. If that information was not available, published mean annual temperatures were used.

### Study index and analyses

A previously published study index, *T*_i_ (Eq. 1), was used in this meta-analysis. This index was designed to measure the N response of trace gas fluxes that may be positive (release from soil) or negative (consumption by soil; Aronson and Helliker, 2010). The standard log-transformed response ratio (Hedges et al., 1999) was not applicable because there were negative average fluxes in some of the studies. In Eq. 1, *C*_i_ and *N*_i_ indicate individual, paired, average measurements of N_2_O fluxes from control (*C*_i_) and N amendment (N_i_) plots.

(1) $$T_{\text{i}}=+∕-\frac{\left|{\text{N}}_{\text{i}}-C_{\text{i}}\right|}{\left|C_{\text{i}}\right|+\left|{\text{N}}_{\text{i}}\right|}$$

The study index is assigned a sign based on the direction of the difference in N_2_O flux due to N amendment: a negative *T*_i_ indicates a decrease in N_2_O release due to N amendment, whereas a positive *T*_i_ indicates an increase in release. The range of *T*_i_ is from −1 to 1, with an index of zero indicating no difference in N_2_O flux between the control and treatment plots. The average of *T*_i_ across multiple studies is given as *T*. The full database of all comparisons and ancillary information is included as Supplementary Material.

Measures of effect size are often weighted with the published variances from the studies included in the analysis. However, in order to increase our sample size, we included studies without associated variances published for each average N_2_O flux value. Although we cannot make conclusions regarding within-study variation, we were able to include nearly three times more studies than similar meta-analyses (Barnard et al., 2005). Since all included studies had performed probability-based statistics and treated their data as normally distributed, we assumed that the distributions of flux responses were normal within each study, and the data altogether were normally distributed.

We used one-way ANOVA, *t*-tests, and Tukey’s honestly significant difference (HSD) to test for differences in categorical variables, and stepwise multiple regression to analyze the impact of multiple continuous variables on effect size. Significance thresholds were *p* < 0.05. All statistical tests were performed using JMP software (Version 9, SAS Institute, Cary, NC, USA).

## Results

Of the 99 comparisons from 33 different studies, 94 showed a larger N_2_O release in the N amended plots relative to control. The exceptions included a temperate deciduous forest in Ambus and Robertson (2006), two comparisons from a temperate grassland in Bijoor et al. (2008), and one from Brown et al. (2012), and a tropical grassland in Steudler et al. (2002). Further, there were two instances where control plots consumed N_2_O, while the rest of the control and N amended plots released N_2_O on average. The locations that consumed N_2_O included a temperate grassland (Flechard et al., 2007) and a temperate needle leaf forest (Jassal et al., 2011).

**Figure 1:**
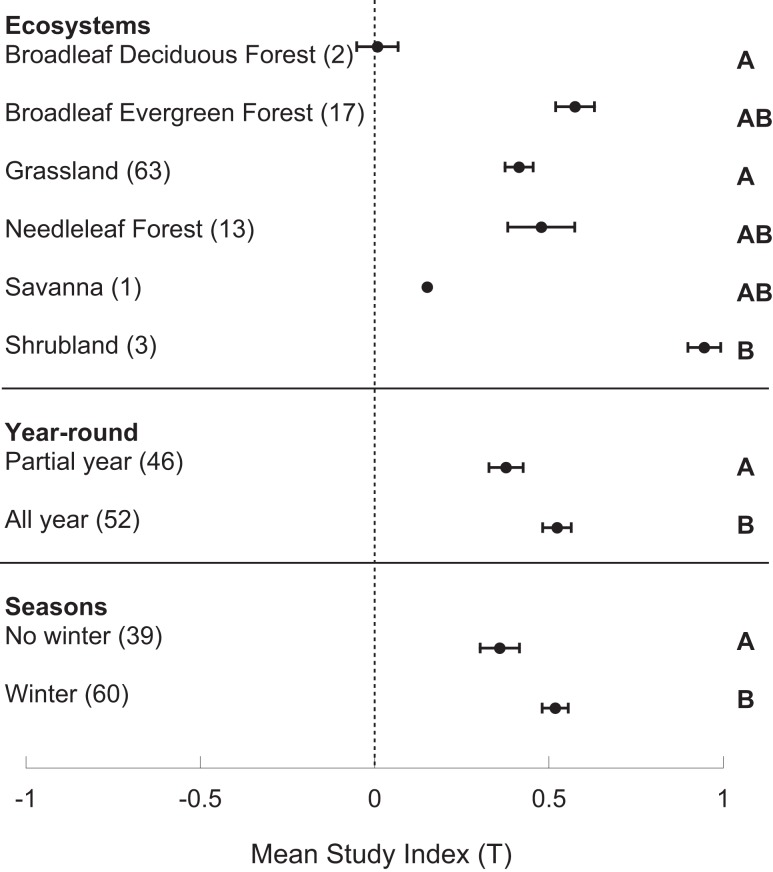
**Estimates of the study index, *T*, in different ecosystems and study time periods**. Means are bounded by the standard error of the mean and letters denote significant differences by Tukey’s HSD.

The study index (*T*) was significantly greater than zero (*p* < 0.001), and varied by ecosystem and season(s) of study. The average ± standard error of *T* was 0.477 ± 0.032. *T* varied by ecosystem (*p* < 0.008), with a larger *T* in shrubland than grassland and broadleaf deciduous forest, but no differences among the other ecosystems (Figure 1). Season of analysis also significantly influenced *T*; studies that included winter months had higher responses to N amendments (*p* < 0.016, Figure 1). Further, *T* was significantly higher in those studies that were performed year-round (*p* < 0.024) as opposed to those only conducted in a subset of seasons (Figure 1). There were no significant differences in *T* by region, gas flux method, duration of N amendment (<5 years versus 5+ years of application), nor timing of N addition (before or during analysis). However, *T* was marginally significantly greater in studies with <5 years of N amendment (*p* < 0.053).

**Figure 2:**
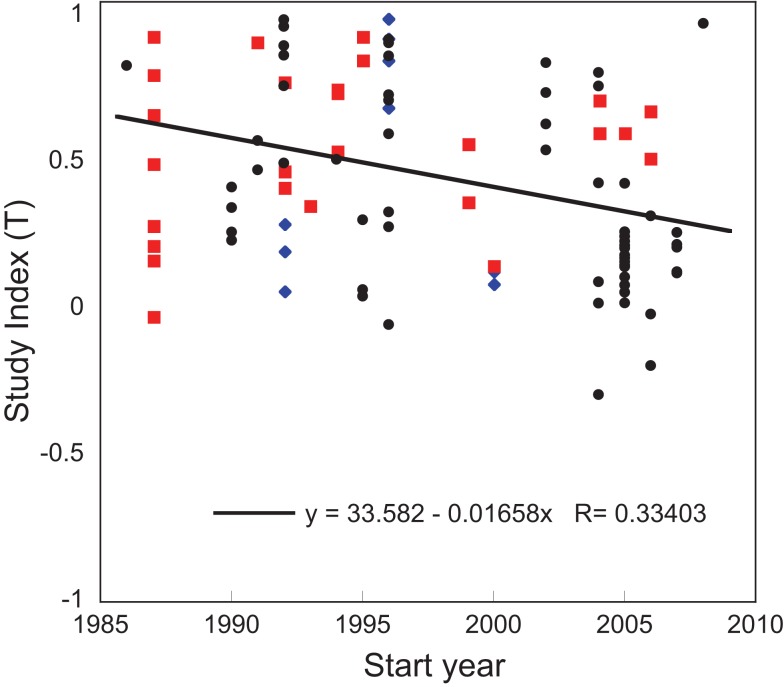
**Regression of study index, *T*, on the start year in control plots**. The temperate sites are shown in back circles, boreal sites are blue diamonds, and the tropical sites are red squares. The regression line is shown for all points.

A stepwise multiple regression of *T* with selected continuous variables revealed that year, amount of N, number of measurements, study duration, precipitation, and latitude all significantly impacted the response to N amendment with a total *R*^2^ of 0.349. The start year of study correlated inversely with *T* (Figure 2), and had the largest partial *R*^2^ (partial *R*^2^ = 0.112). Over the 23 years covered by our analysis, there was a 55% decline in the N_2_O response to N amendment. *T* was also inversely related to study duration (partial *R*^2^ = 0.049). Several variables correlated positively with *T*, including the total amount of N added (partial *R*^2^ = 0.056), the number of measurements (partial *R*^2^ = 0.049), daily precipitation (partial *R*^2^ = 0.025), and latitude (partial *R*^2^ = 0.058).

Further, we investigated whether correlations from the stepwise multiple regression were significant under specific conditions. We found that the negative correlation between *T* and start year of study was driven by the temperate region (*R*^2^ = 0.213, *p* < 0.001). There was no significant relationship in the boreal or tropical regions (*p* < 0.850 and 0.462, respectively). We also found that the positive correlation between daily precipitation and *T* was significant for studies conducted year-round (*R*^2^ = 0.081, *p* < 0.039), but not for studies that omitted seasons (*R*^2^ = 0.001, *p* < 0.830).

## Discussion

Consistent with our hypothesis, we found that N amendment resulted in greater N_2_O release from soils (e.g., Bouwman et al., 2002). This response increased with N amendment level, in contrast with the findings of Barnard et al. (2005) and Lu et al. (2011). However, Barnard et al. (2005) included 11 and Lu et al. (2011) included 8 non-agricultural studies in their analyses, whereas our analysis included 33. Thus we may have observed increasing N_2_O responses to increasing N amendment levels because we included more non-agricultural studies.

Although we did find support for the hypothesis that increasing precipitation correlates with N response, we did not find that shrubland, the driest system, had the lowest response to N. In fact, shrubland had the greatest N_2_O release response, whereas savanna and broadleaf deciduous forest had the lowest. Other forest and grassland ecosystems showed great variation. Although shrublands showed the largest N_2_O response to N amendment, this result was based on only one study, located in subarctic heath (Christensen et al., 1999). Nonetheless, there is some evidence that shrublands in lower latitudes may show similar responses. Soils from a New Mexico shrubland increased N_2_O release rates fourfold under fertilization in laboratory incubations (Crenshaw et al., 2008). Further field-based investigations of arid regions across latitudes should be performed to test this result.

We observed that the effect size resulting from N amendment has decreased over the time period from 1986 to 2008, inclusive. We propose two possible explanations for this trend. One is that methods of quantifying N_2_O changed, but this explanation is not likely because gas chromatography has been the most common method of estimating N_2_O concentrations over the 23 years studied. Further, the type of chamber system used, either static vented or flow-through chambers, was not a significant factor in this analysis. This result is consistent with a previous finding that automated flow-through and manual vented chambers produce similar flux estimates (Smith and Dobbie, 2001).

The second explanation for the observed decrease in response to N amendment over time is that accumulated N deposition has reduced the potential for systems to respond to further N amendment. If N deposition has accumulated over time (Holland et al., 2005), then the observed decrease in response flux may be due to a decrease in the relative difference in N availability between the control and treatment. Longer studies also showed lower N_2_O release responses to N amendment, which suggests the response may diminish as N accumulates in the soil. Further, we found that *T* correlates inversely with the mean N_2_O flux in control plots (*R*^2^ = 0.102, *p* < 0.001), suggesting that N accumulation in control plots could be driving a decrease in *T* over time.

The likelihood that long-term N deposition has reduced the sensitivity of N_2_O release is difficult to evaluate without more information on N deposition history. It is not clear if there has been sufficient cumulative N deposition to suppress the N_2_O response at many of our sites. However, N deposition is high and potentially increasing in certain locations (Holland et al., 2005), such as Oensingen, Switzerland, which receives 15 kg N ha^−1^ year^−1^ (Flechard et al., 2007) and the Kellogg Biological Station LTER in Southwest Michigan, which receives 6 kg N ha^−1^ year^−1^ (Ambus and Robertson, 2006). The correlation between start year and *T* was only significant for the temperate region, which is also the most studied region, and has experienced the highest N deposition rates in recent years (Dentener, 2006). The paucity of study sites in the boreal region may account for the lack of response there, although rates of N deposition are lower there than the temperate zone as well (Holland et al., 2005). However, there were 27 observations in the tropical region, but no correlation. Most tropical soils already have high N availability (Brookshire et al., 2012) and N_2_O release (Park et al., 2011). Therefore accumulated N deposition may not impact the response to additional N amendment. Furthermore, *T* was relatively lower in low (tropical) latitudes, while control fluxes were high, suggesting that these systems are less responsive to additional N.

If the N_2_O response to fertilization declines with increasing cumulative N deposition, then this pattern must be reconciled with the positive response of N_2_O to greater levels of N amendment. Although N_2_O release grows with increasing N amendment in the short term, the long-term response to sustained N addition may be different if ecosystems transition from a state of N-limitation to N-saturation. Adding more N to an already saturated system, which is releasing large amounts of N_2_O, might not stimulate a large increase in N_2_O release. This prediction contrasts with the assertions of Skiba et al. (1999) and Ambus and Robertson (2006); however, those studies were conducted over only a few years. Taken together, our data suggest that N addition in a short time period causes increased N_2_O release, but over time soil microorganisms may change in composition or adapt to increasing N availability, dampening the response to further N deposition increase.

The season of study impacted the response to N amendment. Studies performed year-round had greater effect sizes than those performed in selected seasons, which suggests that many studies may have missed the full magnitude of the response. There was also an impact of season on the environmental response, as only those studies performed year-round captured the response to precipitation. In addition, those studies that included winter were found to have the greatest responses, underscoring the need to assess the response to N amendment year-round. There was also a significant positive correlation between the number of measurements and the response to N amendment, indicating that responses are lower in studies with limited sampling in selected seasons. For processes with high variance, such as N_2_O production, increasing the number of measurements increases the probability of capturing high-flux events.

In conclusion, N amendment increased N_2_O release across studies, with greater rates of N amendment stimulating greater release. The ecosystem with the greatest N_2_O response to fertilization was shrubland, while those with the lowest responses were savannas, and broadleaf deciduous forests. However, our analysis identified only a handful of field-based studies in savanna and shrubland ecosystems, and none in desert ecosystems. Therefore, additional fertilization studies should be performed in these systems. Additional measurements of N deposition should also be performed to determine if the decrease in effect sizes over the past 23 years is due to an accumulation of N deposition, particularly in the temperate region. By decreasing the sensitivity of non-agricultural soils to N addition, sustained N deposition could ultimately dampen the climate feedback of N_2_O release.

## Conflict of Interest Statement

The authors declare that the research was conducted in the absence of any commercial or financial relationships that could be construed as a potential conflict of interest.

## Supplementary Material

The Supplementary Material for this article can be found online at http://www.frontiersin.org/Terrestrial_Microbiology/10.3389/fmicb.2012.00272/abstract

## Appendix

A list of papers from which data are extracted for this metadata analysis.
